# Potential biomarkers of abnormal osseointegration of implants in type II diabetes mellitus

**DOI:** 10.1186/s12903-021-01939-9

**Published:** 2021-11-18

**Authors:** Lingxiao Wang, Zhenhua Gao, Changying Liu, Jun Li

**Affiliations:** 1 Department of Dental Implant Center, Beijing Stomatological Hospital, Capital Medical University, Capital Medical University School of Stomatology, No. 4 Tian Tan Xi Li, Beijing, 100050 People’s Republic of China; 2 Outpatient Department of Oral and Maxillofacial Surgery, Beijing Stomatological Hospital, Capital Medical University, Capital Medical University School of Stomatology, No. 4 Tian Tan Xi Li, Beijing, 100050 People’s Republic of China

**Keywords:** Bioinformatics analysis, Biomarkers, Osseointegration, miRNAs, T2DM

## Abstract

**Background:**

Type II diabetes mellitus (T2DM) is an important risk factor for osseointegration of implants. The aim of this study was to explore key genes of T2DM affecting bone metabolism through bioinformatic analysis of published RNA sequencing data, identify potential biomarkers, and provide a reference for finding the molecular mechanism of abnormal osseointegration caused by T2DM.

**Methods:**

We identified differentially expressed mRNAs and miRNAs from the Gene Expression Omnibus database using the R package ‘limma’ and analysed the predicted target genes using Kyoto Encyclopedia of Genes and Genomes pathway enrichment analysis and Gene Ontology analysis. At the same time, miRNA–mRNA interactions were explored using miRWalk 2.0.

**Results:**

We constructed an miRNA-gene regulatory network and a protein–protein interaction network. The enrichment pathways of differentially expressed mRNAs included extracellular matrix receptor interactions, protein digestion and absorption, the PI3K-Akt signalling pathway, cytokine–cytokine receptor interactions, chemokine signalling pathways, and haematopoietic cell lineage functions. We analysed the expression of these differentially expressed mRNAs and miRNAs in T2DM rats and normal rats with bone implants and identified *Smpd3*, *Itga10*, and *rno-mir-207* as possible key players in osseointegration in T2DM.

**Conclusion:**

*Smpd3*, *Itga10*, and *rno-mir-207* are possible biomarkers of osseointegration in T2DM. This study sheds light on the possible molecular mechanism of abnormal osseointegration caused by bone metabolism disorder in T2DM.

**Supplementary Information:**

The online version contains supplementary material available at 10.1186/s12903-021-01939-9.

## Background

With the development of dental implant technology and materials, implant-supported prostheses have become the first choice for restoring oral function and aesthetics among patients with missing teeth. Type II diabetes mellitus (T2DM) is a chronic disease; it has a high prevalence and can have adverse effects on tissues and organs throughout the body [1–3]. T2DM is an important risk factor for periodontal disease and alveolar bone loss. The rate of tooth loss in T2DM patients is 15% higher than that in healthy people, and the proportion of T2DM patients with dental implants is increasing [4, 5]. Regarding implant osseointegration, the biological function of alveolar bone-derived cells, the blood supply of the alveolar bone, and the metabolic status of bone tissue all play important roles in implant osseointegration. Previous studies have shown that the proliferation and osteogenic differentiation abilities of osteoblasts are weaker in patients with diabetes mellitus than in healthy people. Human blood carries a large amount of active protein and oxygen that can affect the state of bone-derived cells around the implant, and T2DM is often accompanied by high levels of glucose [6, 7]. Patients with diabetes often have accompanying calcium and phosphorus metabolism disorders, which affect bone tissue remodelling and interfere with implant-bone bonding, especially 1–3 weeks after implant placement. Bone tissue remodelling disorders caused by diabetes are important risk factors for the failure of oral implants [8–10].

At present, identifying the molecular mechanisms underlying bone tissue remodelling disorders around dental implants in diabetes patients is an area of active research. Several studies have shown that microRNAs (miRNAs) play an important role in the occurrence and development of bone tissue remodelling around dental implants [11–14]. miRNAs are single-stranded non-coding RNA molecules with approximately 20–24 nucleotides. They are highly evolutionarily conserved and are mainly involved in post-transcriptional regulation. Recent studies have confirmed that miRNAs can inhibit mRNA expression or reduce mRNA stability by specifically binding to the 3′-untranslated regions of target mRNAs, thus, regulating the expression of related genes. miRNAs play an important role in cell proliferation, differentiation, and apoptosis, and some studies have confirmed that several miRNAs are involved in osteogenic differentiation and bone metabolism-related diseases that affect bone tissue remodelling and new bone formation [15–18].

miRNA–mRNA networks have been widely used to study pathways of various cancers and cardiovascular and neurodegenerative diseases [19–27]. However, miRNA–mRNA networks have not been assessed when determining how diabetes affects implant osseointegration. The main difficulty stems from the fact that implant osseointegration is a biological process in which bone tissue and the implant’s titanium metal surface directly come into contact to produce a stable combination. The effects of the implant are continuously transmitted and dispersed in the bone tissue, in terms of not only bone formation but also physiological bone absorption and bone maturation. At the cellular level, this mainly involves the multidirectional regulation of osteoblasts, osteoclasts, and macrophages, among other cell types. Accurately manipulating the molecular regulatory network while in the process of implant-bone binding is key to constructing a miRNA–mRNA network for this process [28–34]. One study found that when inserting an implant in bone, a controlled partial fracture is created, and the fracture healing process is very similar to that following implant placement. In both osseointegration and fracture healing, substantial bone formation is followed by bone resorption and maturation [35]. Overall, the purpose of this study was to identify miRNAs and mRNAs that could be used as potential predictive biomarkers or therapeutic targets for bone metabolism disorders caused by T2DM. Therefore, in this study, we used callus tissue to identify differentially expressed (DE) mRNAs and miRNAs between rats and those with T2DM. Moreover, we used miRWalk 2.0 to study miRNA–mRNA interactions, established an miRNA–mRNA network, and constructed an implantation model of T2DM rats to verify the DE miRNAs and mRNAs.

## Methods

### Collection of RNA-seq datasets

The Gene Expression Omnibus (GEO, https://www.ncbi.nlm.nih.gov/geo) database was searched to identify all datasets that evaluated mRNA and miRNA expression in bone tissue samples of T2DM animal models. The following terms from the medical subject headings thesaurus were used for the search: (“diabetes” OR “diabetic” OR “T2DM” OR “diabetes mellitus”) AND (“miRNA” OR “mRNA”) AND (“bone tissue”). Finally, two RNA sequencing (RNA-seq) datasets, GSE76364 and GSE76365, were obtained. The details of GSE76364 and GSE76365 are included in Additional file 1: Table S1. GSE76364 only contained mRNA expression data from the bone callus of T2DM rats (*Rattus norvegicus*), including 10 T2DM animals and 10 healthy animals. GSE76365 only contained miRNA expression data from the same tissue of the rats.

### Identification of DE mRNAs and miRNAs

The ENSEMBL IDs of samples were converted by annoE 1.0.3 (https://github.com/ChrisLou-bioinfo/AnnoENSG2GENE), based on GENCODE 31 (19.06.19) [36]. Differential expression analysis was performed using edgeR, an R package for differential expression analysis of biological RNA-seq replicates to identify significant DE mRNAs and miRNAs in samples from T2DM rats. All q-values were corrected for multiple testing according to false discovery rate (FDR). DE mRNAs with an absolute log_2_FC > 2 and FDR < 0.05, and DE miRNAs with an absolute log_2_FC > 1 and FDR < 0.05 were considered significant and were visualized using volcano plots.

### Gene ontology (GO) and kyoto encyclopedia of genes and genomes (KEGG) analysis of DE mRNAs

Significant DE mRNAs were analysed for GO and KEGG enrichment using Omicshare (https://www.omicshare.com). GO was used to describe gene functions across three categories: the biological process (BP), cellular component (CC), and molecular function (MF). Findings from GO and KEGG analyses were searched for results at the significance level set at an adjusted *P* value of < 0.05.

### miRNA–mRNA targeting relationship prediction

The miRNA–mRNA interactions were predicted by miRWalk 2.0, which incorporates 12 algorithms for prediction (TargetScan, RNAhybrid, RNA22, PITA, PicTar2, miR-Walk, Microt4, miRNAMap, miRDB, mirBridge, miRanda, and miRMap) [37]. The miRNA–mRNA interactions were visualized using Cytoscape 3.7.1 [38].

### KEGG analysis for target mRNAs

Omicshare (https://www.omicshare.com) was used for KEGG analysis of the target mRNAs. The KEGG analysis results were searched for pathways at the significance level adjusted at *P* < 0.05.

### Construction of an miRNA–mRNA regulatory network

Based on the analysis of DE miRNAs, DE mRNAs, and enrichment pathways, Cytoscape 3.7.1 was used to visualize the miRNA–mRNA regulatory network.

### Ethical statement

All experiments on animals were performed in accordance with the ARRIVE guidelines 2.0 and the Ethical Guidelines for the Care and Use of Laboratory Animals of the United States National Institutes of Health. The study protocol, including procedures for animal handling and husbandry, was reviewed and approved by the Animal Experiments and Experimental Animal Welfare Committee of Capital Medical University, Beijing, China (protocol number 2017-104).

### Animals and treatment

Ethical approval was granted by the Animal Care and Use Committee of Capital Medical University (approval number 2017-104). All animal experiments were conducted according to the ethical principles of the Animal Care and Use Committee of Capital Medical University. Twenty 2-week-old male Wistar rats (average weight of 300 ± 20.0 g) were purchased from SPF (Beijing) Biotechnology (Beijing, China). They were randomly divided into an experimental group (n = 10) and a control group (n = 10). The sample size was calculated according to the following formula:

$${\text{n}} = \frac{{\left( {Z_{ \alpha } + Z_{ \beta } } \right)^{2} *2 \sigma^{2} }}{{\delta^{2} }}.$$

The rats were housed under controlled conditions (25 °C and a 12-h light/dark cycle) and had ad libitum access to water and rat chow (SPF [Beijing] Biotechnology). The experimental group was fed with diabetes-specific induction feed (SBF [Beijing] Biotechnology) for 7 weeks and were then given a single intraperitoneal injection (40 mg/kg) of streptozotocin (STZ). Fasting blood glucose was measured on days 0, 3, 6, 9, 12, 15, 18, 21, 24, 27, and 30 after STZ treatment. Three days after STZ treatment, animals with fasting blood glucose levels ≥ 16.7 mmol/L were considered to have diabetes (Additional file 1: Figure S1) [39]. A group of vehicle control rats were injected with an equal volume of 0.1 M citrate buffer. The left leg of each rat was shaved and disinfected. Sterile gauze was laid on the left leg, and the skin was cut approximately 5 mm from the proximal end of the femoral metaphysis with a diameter of 1 mm and a depth of 2 mm by using a minimally invasive approach with water cooling to prepare it for implant placement. After the operation, all rats received a subcutaneous injection of 0.5 mg/kg ketoprofen.

### Euthanasia procedures for experimental animals and sampling technique

We anaesthetised the animals with 12 mg/kg of xylazine and 80 mg/kg of ketamine via intraperitoneal injection and then euthanised them by exsanguination after 7, 14, or 21 days. Under anaesthesia, the abdominal aortas of rats were punctured to collect 8 mL of blood into heparinised tubes. The leg bone with the implant was collected, the bone tissue around the implant was carefully removed with a wire saw and implant motor (ensuring that 1 mm of bone tissue remained around the implant); we then carefully scraped off the 1 mm of bone tissue around the implant with a scraper and put it into TRIzol reagent (Invitrogen, Carlsbad, CA). After that, the animal remains were disposed of in an incinerator.

### RT-qPCR analysis

For analysis of mRNA levels, total RNA from the 1-mm layer of femur tissues around implants were prepared and isolated using TRIzol reagent (Invitrogen). Complementary DNA was synthesised using the RevertAid First Strand Complementary DNA Synthesis kit (Thermo Fisher Scientific, Waltham, MA). Quantitative real-time PCR was performed using the SYBR Premix Ex TaqII (Takara, Dalian, China), as per the manufacturer’s instructions. U6 and *GAPDH* were used as internal controls. Relative expression was calculated using the 2^−ddCt^ method. The primer sequences used are shown in Additional file 1: Table S2.

### Receiver operating characteristic (ROC) curves

ROC curves were constructed to distinguish DE hub genes and miRNAs in the plasma of T2DM rats and normal rats, and the areas under the ROC curves (AUCs) were analysed to evaluate the diagnostic accuracy. All statistical analyses were performed using SPSS 13.0 (IBM, Armonk, NY).

### Statistical analyses

A Student’s *t* test was used to compare test and control samples in GraphPad Prism 7 (GraphPad Software, San Diego, CA). Data are presented as means ± standard deviations for quantitative variables. Mean values of quantitative variables were evaluated by a Student’s *t* test or the Mann–Whitney U test when the requirements for a Student’s *t* test were not met. A *P* value of ≤ 0.05 was considered statistically significant. All statistical analyses were performed using SPSS 13.0 (IBM).

## Results

### Heterogeneity test and preliminary screening of DE mRNAs and miRNAs

**Fig. 1:**
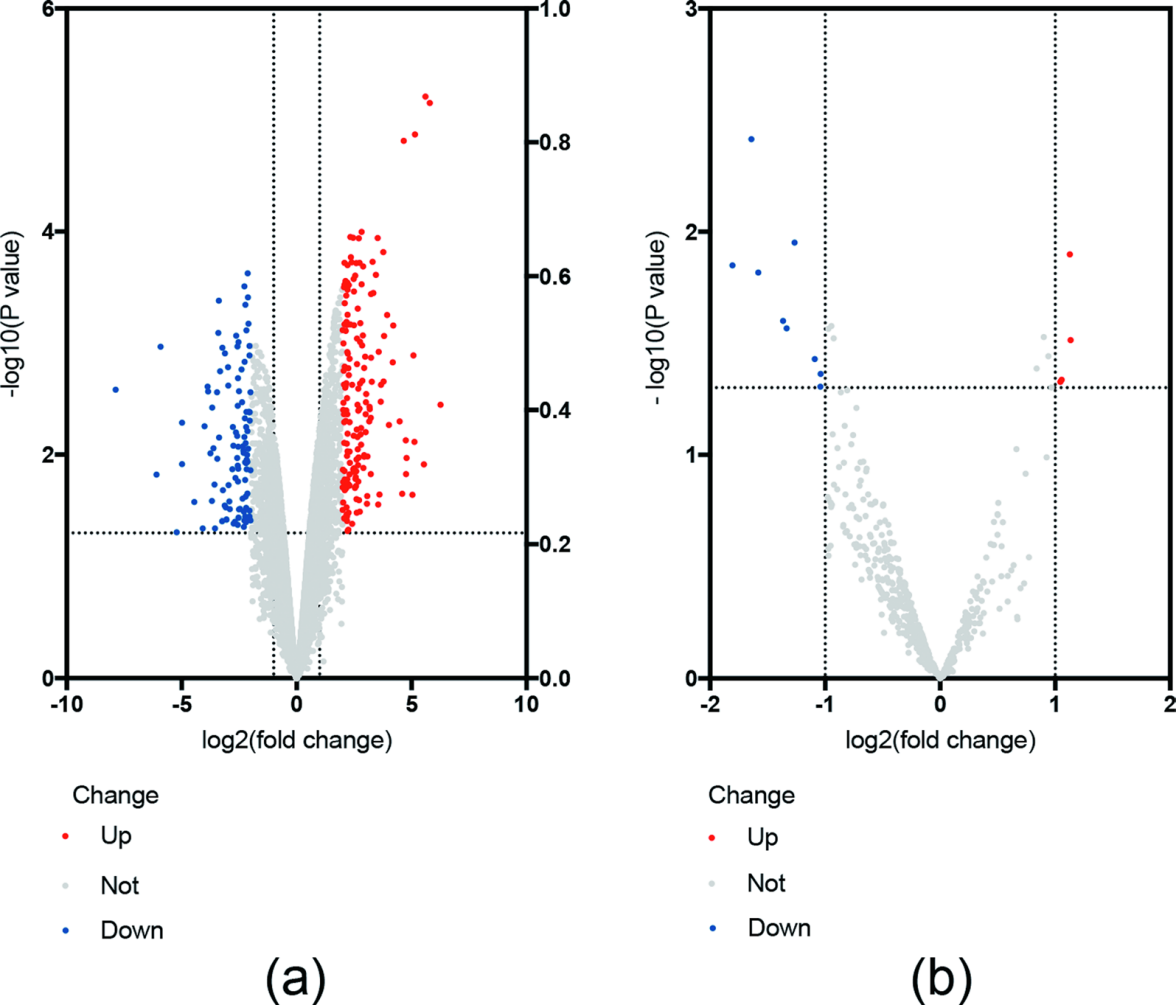
The volcano plot of significant differentially expressed mRNAs and miRNAs in GSE76364 and GSE76365 (**a**) and GSE76364 (**b**). Red dots represent upregulated mRNAs and miRNAs, and blue dots represent downregulated mRNAs and miRNAs

Two RNA-seq datasets (GSE76364 and GSE76365) were included in our study. These comprised 280 significant DE mRNAs, including 173 upregulated and 107 downregulated mRNAs, in GSE76364 (Fig. 1a), and 13 significant DE miRNAs in GSE76365, including 4 upregulated and 9 downregulated miRNAs (Fig. 1b).

### GO and KEGG analyses of significant DE mRNAs

**Fig. 2:**
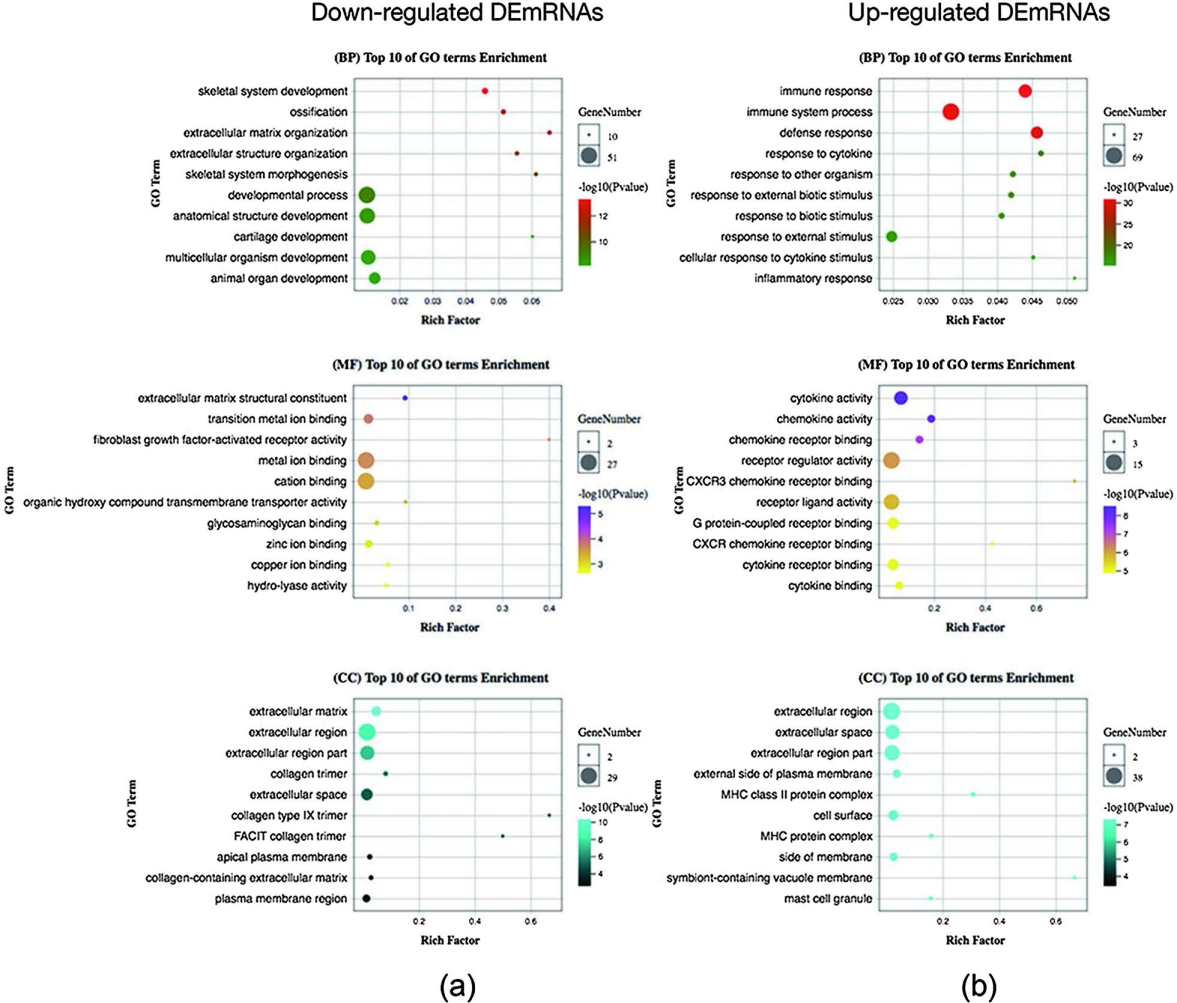
Gene Ontology (GO) analyses for significant differentially expressed mRNAs in GSE76364. The bubble charts present GO analysis results of downregulated (**a**) and upregulated mRNAs (**b**)

The significantly upregulated and downregulated DE mRNAs of GSE76364 were utilized for GO and KEGG analyses. On analysing the GO terms of the GSE76364 dataset, the three most enriched BP terms of the downregulated DE mRNAs were “skeletal system development,” “ossification,” and “extracellular matrix organization,” and the three most enriched BP terms of the upregulated DE mRNAs were “immune response,” “immune system process,” and “defence response.” The three most enriched MF terms of the downregulated DE mRNAs were “extracellular matrix structural constituents,” “transition metal ion binding,” and “fibroblast growth factor-activated receptor activity cytokine activity,” and the most enriched MF terms of the upregulated DE mRNAs were “chemokine activity” and “chemokine receptor binding.” The three most enriched CC terms of the downregulated DE mRNAs were “extracellular matrix,” “extracellular region,” and “extracellular region part,” and the three most enriched CC terms of the upregulated DE mRNAs were “extracellular region,” “extracellular space,” and “extracellular region part” (Fig. 2).

**Fig. 3:**
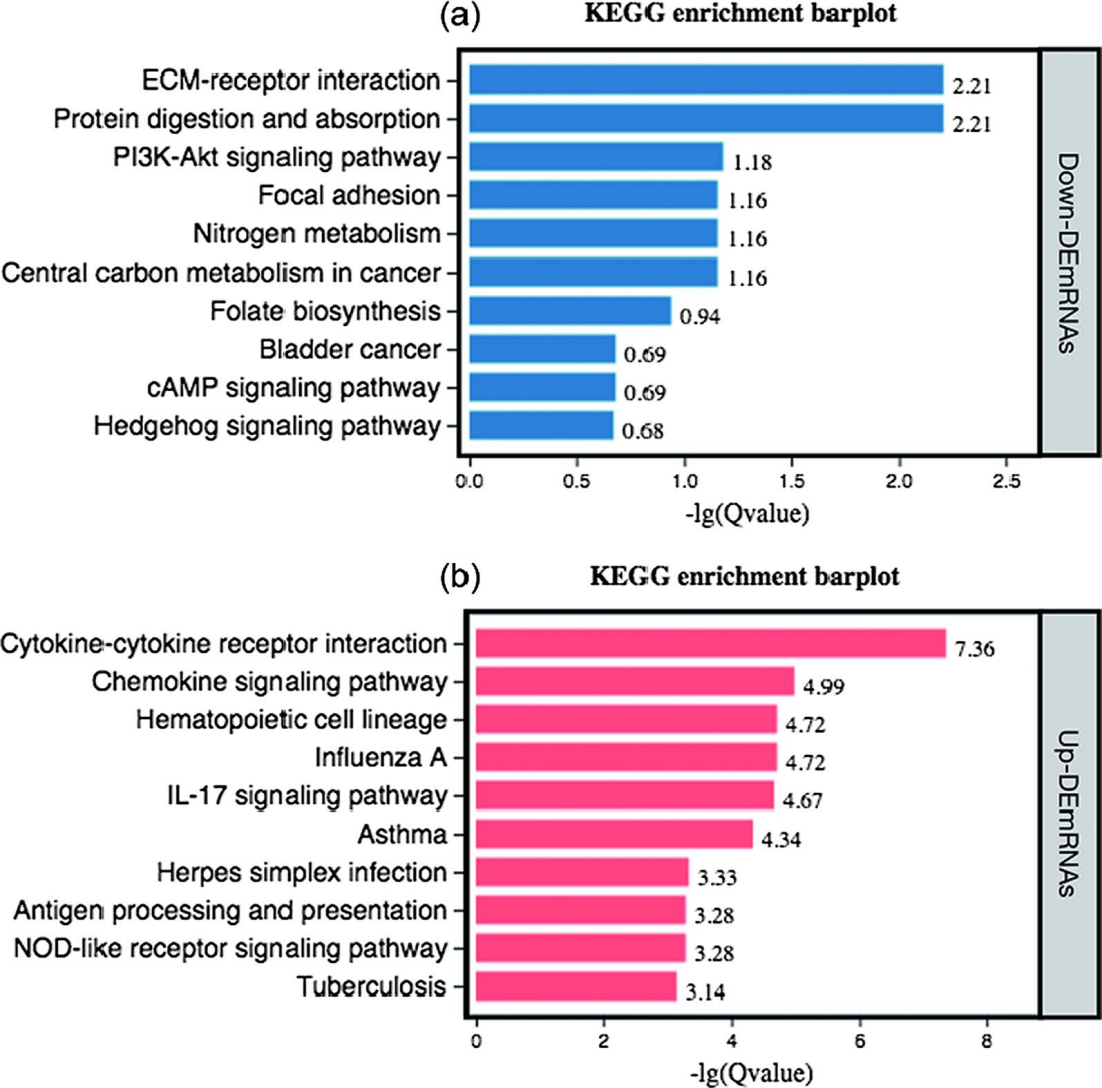
Kyoto Encyclopedia of Genes and Genomes (KEGG) analyses for significant differentially expressed mRNAs in GSE76364. The bar charts present KEGG analysis results of downregulated (**a**) and upregulated mRNAs (**b**)

In the KEGG pathway enrichment analysis of the GSE76364 dataset, extracellular matrix (ECM)-receptor interaction, protein digestion and absorption, and the PI3K-Akt signalling pathway were enriched in downregulated DE mRNAs. In the upregulated DE mRNAs, cytokine–cytokine receptor interaction, chemokine signalling pathways, and haematopoietic cell lineage functions were the three most enriched pathways (Fig. 3).

### DE miRNA target gene prediction

**Fig. 4:**
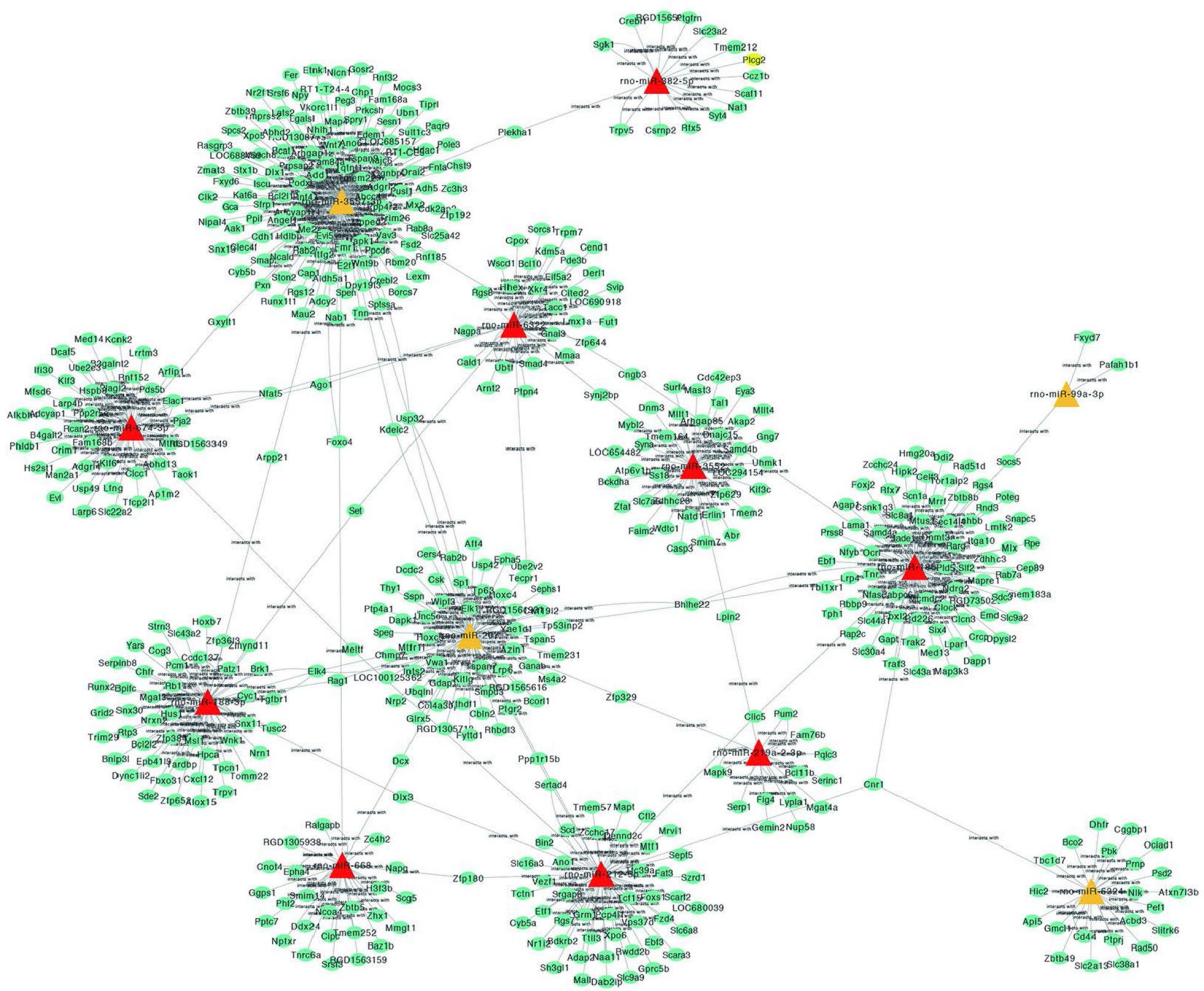
miRNA–mRNA regulatory network. The relationship between 13 key miRNAs and 719 target genes. Triangle, miRNAs. Ellipse, genes. Yellow, overexpression. Red, downregulation. Straight lines, regulatory relationship between miRNAs and genes

We constructed an miRNA–mRNA regulatory network of 13 miRNAs (four upregulated, nine downregulated; Additional file 1: Table S3) and 719 predicted target genes. The number of genes around an miRNA or the number of miRNAs around a surrounding gene was defined as degrees: the more important the network, the greater the degree of its base. Three miRNAs, including *miR-3557-3p* (degree = 178), *miR-185-3p* (degree = 97), and *miR-207* (degree = 80), were the core regulatory network miRNAs (Fig. 4).

### Intersecting DE miRNA target genes with DE mRNAs and constructing a protein–protein interaction (PPI) network

**Fig. 5:**
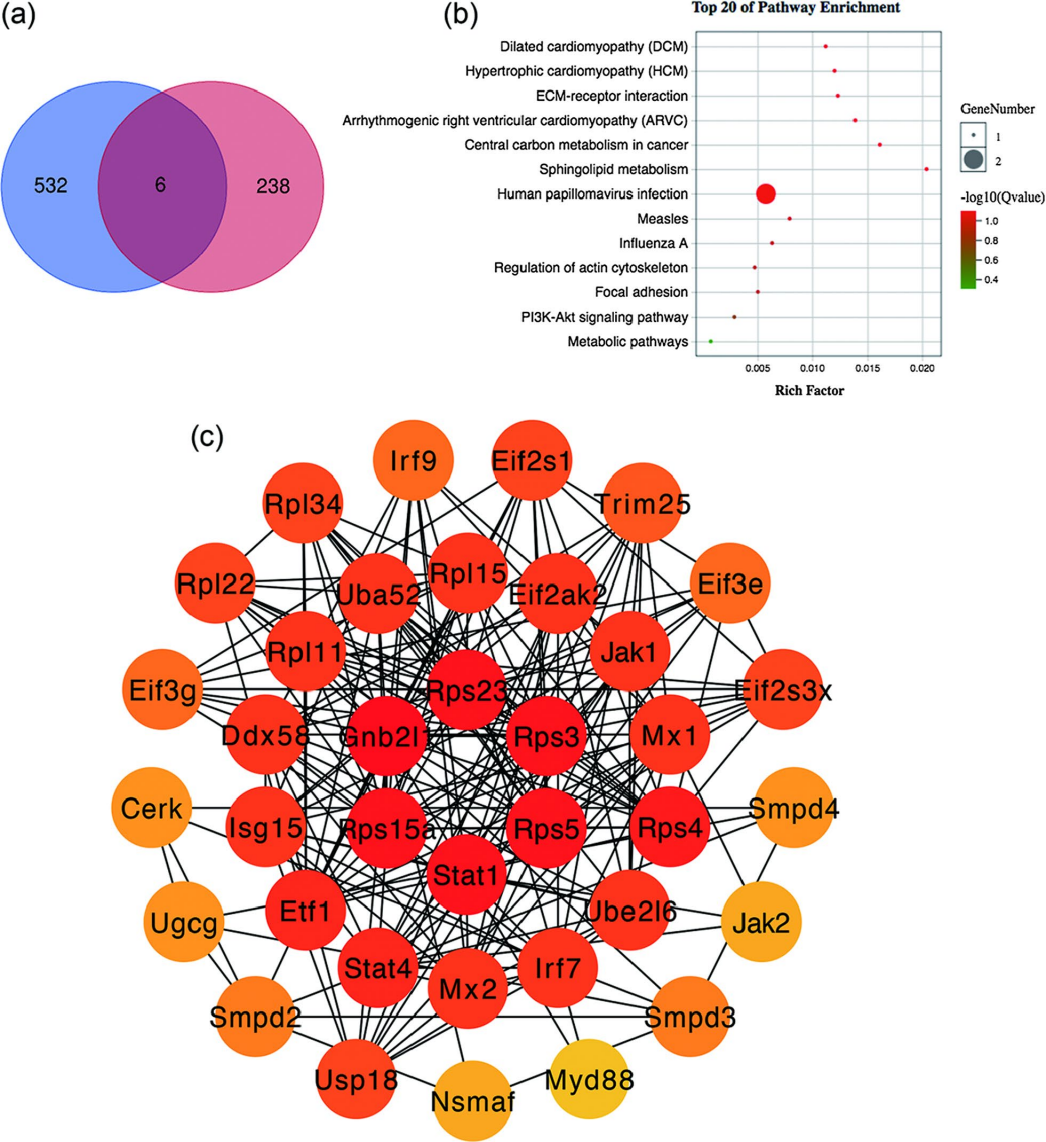
The six key differentially expressed (DE) mRNAs and protein–protein interaction network. The significant DE mRNAs of GSE104674 and the 13 miRNA-targeted mRNAs are visualized by a Venn plot (**a**). Kyoto Encyclopedia of Genes and Genomes (KEGG) analyses for six significant DE mRNAs (**b**). Circles represent genes, and darker colours represent larger degrees. Protein–protein interaction network (**c**)

The six intersecting genes, including *Smpd3*, *Itga10*, *Slc16a3*, *Gca*, *Xkr4*, and *Mx2* (Additional file 1: Table S4), were obtained by intersecting the 280 DE mRNAs with 719 target mRNAs (Fig. 5a). In the KEGG pathway enrichment analysis of the six genes, dilated cardiomyopathy, hypertrophic cardiomyopathy, and ECM-receptor interaction were the three most enriched pathways (Fig. 5b). Using the STRING database to predict gene interactions, we set a high confidence level (0.700) for the minimum required interaction score and constructed a PPI network (Fig. 5c) of the DE genes in Cytoscape. The average node degree was 12.7, and the top six degrees were *Gnb2l1* (degree = 19), *Stat1* (degree = 15), *Rps15a* (degree = 15), *Rps23* (degree = 15), *Rps3* (degree = 15), and *Rps5* (degree = 15).

### Validation of the identified miRNAs and mRNA

**Fig. 6:**
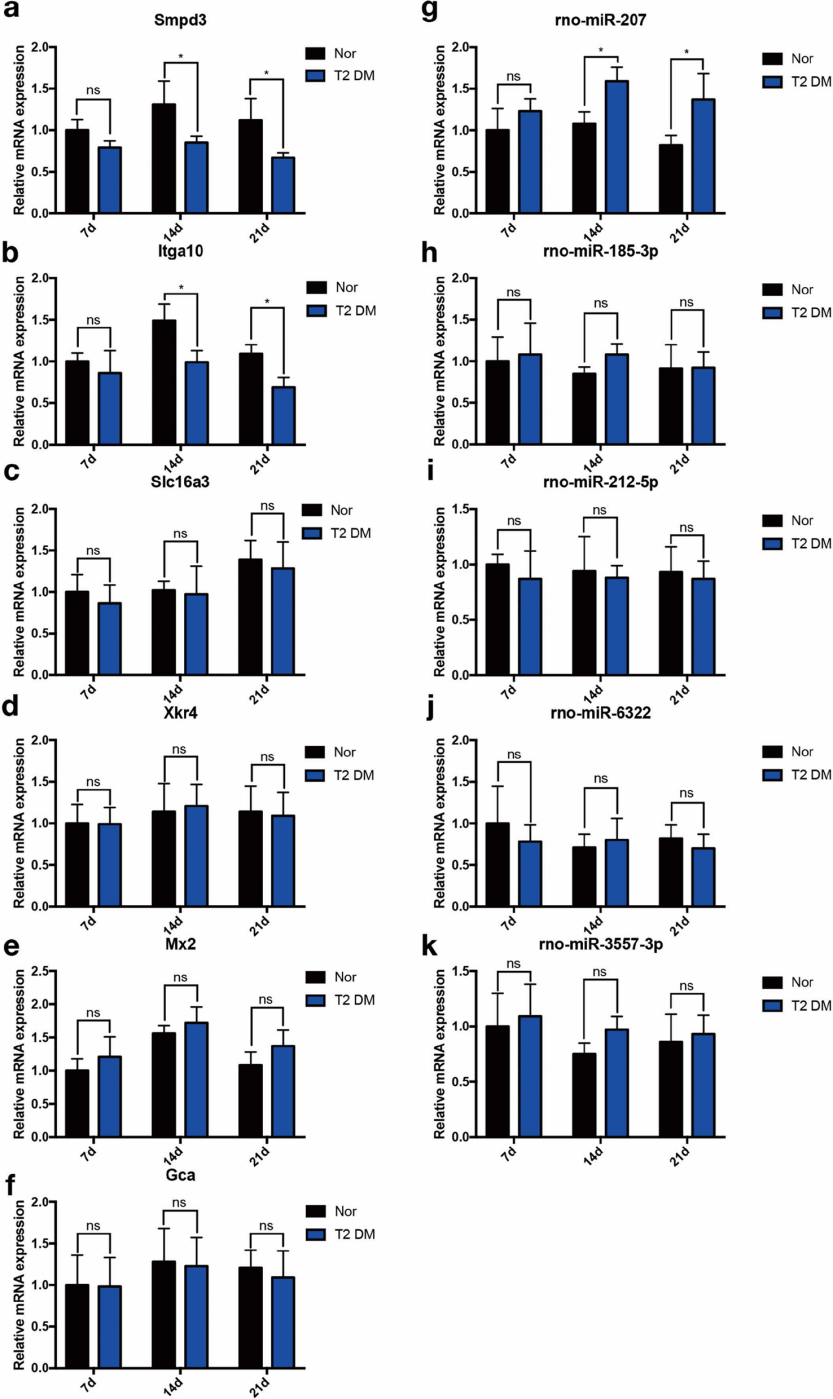
The relative expression of differentially expressed miRNA and mRNA in bone tissue after implanting 7, 14, and 21 days between T2DM and normal rats. **a** *Smpd3*. **b** *Itga10*. **c** *Slc16a3*. **d** *Xkr4*. **e** *Mx2*. **f** *Gca*. **g** *rno-miR-207*. **h** *rno-miR-185-3p*. **i** *rno-miR-212-5p*. **j** *rno-miR-6322*. **k** *rno-miR-3557-3p*. *P < 0.05. *T2DM* type II diabetes mellitus

**Fig. 7:**
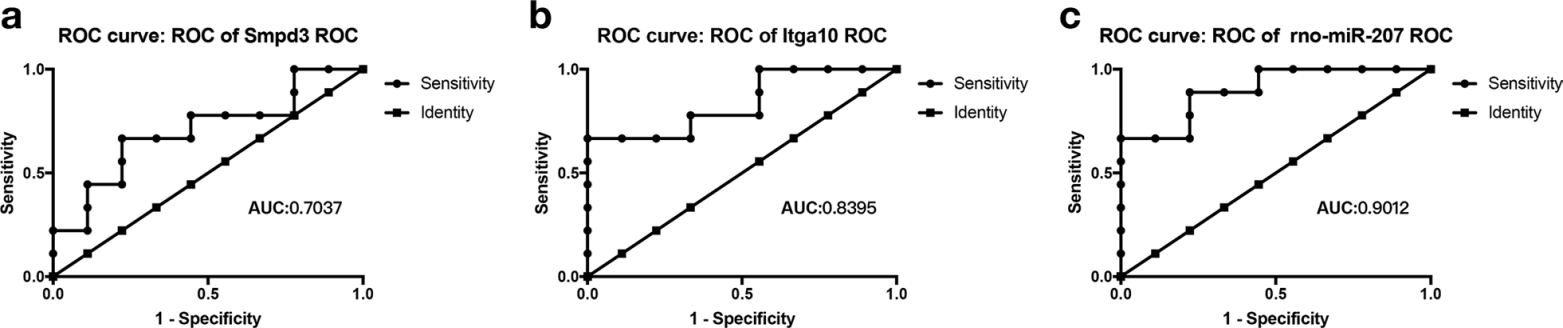
Receiver operating characteristic curves (ROC) of *Smpd3*, *Itga10*, and *rno-mir-207* between T2DM and normal rats. **a** *Smpd3*, **b** *Itga10*, **c** *rno-miR-207*. *T2DM* type II diabetes mellitus

During the study period, none of the animals died. miRNA and mRNA levels in the implants were evaluated by quantitative PCR. As shown in Fig. 6, the expression of *Smpd3* and *Itga10* in the T2DM group decreased 14 and 21 days after implantation, respectively, while the expression of *rno-mir-207* increased (Fig. 6a–k). To study the effectiveness of *Smpd3*, *Itga10*, and *rno-mir-207* as potential biomarkers of T2DM-induced abnormal bone binding, we analysed the ROC curves of these genes in T2DM and normal rats. The AUC values of *Smpd3*, *Itga10*, and *rno-mir-207* were 0.7037, 0.8395, and 0.9012, respectively (Fig. 7a–c), which were all greater than 0.7. An AUC value higher than 0.7 indicates high accuracy [40]. These results indicate that *Smpd3*, *Itga10*, and *rno-mir-207* have the potential for clinical application.

## Discussion

In 2017, approximately 451 million patients worldwide were reported to have diabetes, and this number is estimated to increase to over 592 million by 2035. With the increase in the prevalence of diabetes, its various complications will impose a large economic burden on the world [41, 42]. In the field of dental implantology, it has been confirmed that diabetes can cause metabolic disorders of the bone tissue, ultimately leading to failure in implant osseointegration [43–47]. In recent years, with the development of basic research on T2DM and its related metabolic diseases, miRNAs have shown great potential as biomarkers of bone tissue metabolism-related diseases [48–51]. To further study the biological effects of miRNA–mRNA interactions on bone metabolism in patients with diabetes, we constructed an miRNA–mRNA network, including 13 miRNAs and 6 key mRNAs. In addition, we established a rat model of T2DM, verified the hub genes and target miRNAs, and found that *Smpd3*, *Itga10*, and *rno-mir-207* may be potential biomarkers of abnormal bone binding induced by T2DM.

In our study, we used a multi-step method to identify DE mRNAs in callus tissue from T2DM rats and performed functional and pathway enrichment analysis. The significantly enriched pathways included ECM–receptor interactions, protein digestion and absorption, the PI3K-Akt signalling pathway, cytokine–cytokine receptor interactions, chemokine signalling pathways, and haematopoietic cell lineage functions. The PI3K-Akt signalling pathway plays an important role in bone tissue metabolism, as it can promote the proliferation and differentiation of osteoblasts and participate in the signalling of downstream nuclear factor kappa B receptor activating factor and macrophage colony stimulating factor receptor. Thus, this pathway plays an important role in osteoclast differentiation and survival, and bone resorption, and inhibiting PI3K-Akt signalling pathway activity will weaken osteoclastic bone resorption [52, 53]. In addition, the PI3K-Akt-mTOR is a classical pathway in response to insulin signalling. After food consumption, decomposed glucose enters the blood to promote the release of insulin. Insulin, as a signal of nutrient surplus, guides cells to absorb and utilise these nutrients. Insulin first binds to cell-surface receptors and activates the PI3K-Akt pathway through IRS1. Akt directly promotes the absorption of glucose. At the same time, it activates the activity of mTORC1 through Akt-TSC1/2-Rheb-mTORC1. mTORC1 further guides protein synthesis and uses enzymes related to glucose biosynthesis for nutritional storage [54].

miRNAs can regulate gene expression by inhibiting the translation of target genes or by reducing their stability at the post-transcriptional level. One-third of the human genome can be regulated by miRNAs [55]. Among the selected miRNAs, *mir-99a-3p* can regulate bone homeostasis and osteoblast differentiation [56]; *mir-382-5p* is closely related to the decrease of bone mineral density caused by T2DM [57]; *miR-185* depletion promotes osteogenic differentiation and suppresses bone loss in osteoporosis through the BGN-mediated BMP/SMAD pathway [58]; and *mir-188* can inhibit cell formation, promote fat accumulation in the bone marrow, and it significantly increases in elderly patients [59]. The regulation of cell function is the key to bone formation on the surface of intraosseous implants. As a bridge, non-coding RNAs, especially miRNAs, are directly connected with bone binding as a key factor to control cell function, highlighting the important role of miRNAs in bone binding control. Some emerging in vitro and in vivo studies have emphasised the role of miRNA in bone integration. Many miRNAs affect key bone induction pathways that control OSX, Runx2, and BMP/Smad function, while others affect the monocyte/macrophage lineage. Although great progress has been made in clarifying the mechanism related to the regulation of osteoblast differentiation by miRNAs, those related to bone integration have not been adequately identified and characterised.

In addition, based on RT-qPCR data and ROC analysis, we identified two important hub genes (*Smpd3* and *Itga10*) and an miRNA (*rno-mir-207*). *Smpd3* is expressed in bone and cartilage, and its deficiency leads to cartilage dysplasia and poor bone mineralisation [60–62]. ITGA10 is a transmembrane protein, which is mainly involved in cell adhesion and signal transduction on the cell surface. Studies have shown that ITGA10 can regulate the balance between osteogenesis and osteoclastogenesis and then rescue glucocorticoid-induced osteoporosis [63]. The miRNA *rno-mir-207* is downregulated after osteogenic differentiation of bone marrow-derived stromal cells induced by tacrolimus, suggesting that it may play an important regulatory role in osteogenic differentiation of bone marrow-derived stromal cells [64]. Together, these studies suggest that *Smpd3*, *Itga10*, and *rno-mir-207* may have potential regulatory effects on bone metabolism, but miRNA and mRNA verification is new in the field of oral implant osseointegration and the potential roles of these genes in bone metabolism are unclear. Compared with microarray data, bioinformatics analysis appears to be a promising approach, and it is advantageous as it can create networks using genes, protein–protein interactions, pathway enrichment, and miRNA–mRNA interactions. Here, we show its utility in studying the molecular mechanism of bone tissue metabolism disorder in T2DM.

Our study has some limitations. In the present study, we established a T2DM animal model using the classic STZ injection method. This method is highly repeatable, efficient, and relatively cheap. However, GK rats are a non-insulin-dependent spontaneous T2DM model, which mainly manifests in the islets of Langerhans. These rats feature impaired secretion of β cells; increased glycogen production; insulin resistance induced by liver, muscle, and adipose tissue; and various non-obesity-related complications of diabetes, which are all closely related to human T2DM. In addition, the function and molecular mechanisms of genes are complex. Therefore, the genes identified in this study still need to be verified by cell and animal experiments.

## Conclusions

We created an miRNA–mRNA network to further understand the effects of diabetes on bone metabolism. In this network, miRNAs target and regulate the expression of mRNAs, and this affects the occurrence and development of metabolic disorders in the bone tissue of patients with T2DM. Moreover, in this study, we found that *Smpd3*, *Itga10*, and *rno-mir-207* may be potential biomarkers of T2DM affecting osseointegration. The results of this study can provide a reference for risk assessment and intervention targets when placing oral implants and in developing further clinical applications of this approach.

## Supplementary Information

**Additional file 1.** Additional information.

## Data Availability

The data sets used and/or analysed during the current study are available from the corresponding author on reasonable request. Microarray data are deposited in the Gene Expression Omnibus database under the accession code GSE76364 and GSE76365.

## Abbreviations

T2DM: Type II diabetes mellitus
RT-qPCR: Quantitative real-time PCR
mRNA: Messenger RNA
miRNAs: MicroRNAs
PI3K: Phosphatidylinositol 3-kinase
DE: Differentially expressed
GEO: Gene Expression Omnibus
GO: Gene Ontology
KEGG: Kyoto Encyclopedia of Genes and Genomes
BP: Biological process
CC: Cellular component
MF: Molecular function
STZ: Streptozotocin
ROC: Receiver operating characteristic
AUC: Area under the receiver operating characteristic curve
ECM: Extracellular matrix
PPI: Protein–protein interaction
*Smpd3*: Sphingomyelin phosphodiesterase 3
*Itga10*: Integrin subunit alpha 10
*Slc16a3*: Solute carrier family 16 member 3
*Gca*: Grancalcin
*Xkr4*: XK-related 4
*Mx2*: MX dynamin like GTPase 2
rno: Rattus norvegicus
BMP: Bone morphogenetic protein
