# Supplementary material for: Potential biomarkers of abnormal osseointegration of implants in type II diabetes mellitus
Source: BMC Oral Health. 2021 Nov 18;21:583. doi: 10.1186/s12903-021-01939-9 (PMC8603511; doi:10.1186/s12903-021-01939-9)
Supplement: Supplementary file 1 — Additional file 1. Additional information. [file 12903_2021_1939_MOESM1_ESM.docx]

Potential biomarkers of abnormal osseointegration of implants in type II diabetes mellitus

Ling-xiao Wang^1^, Zhen-hua Gao^2^, Changying Liu^1^, and Jun Li^1^*

^1^ Department of Dental Implant Center, Beijing Stomatological Hospital, Capital Medical University, Capital Medical University School of Stomatology, No. 4 Tian Tan Xi Li, Beijing 100050, China

^2^ Outpatient Department of Oral and Maxillofacial Surgery, Beijing Stomatological Hospital, Capital Medical University, Capital Medical University School of Stomatology, No. 4 Tian Tan Xi Li, Beijing 100050, China

*Corresponding Author:

Jun Li

Department of Dental Implant Center, Beijing Stomatological Hospital

Capital Medical University, Capital Medical University School of Stomatology

No. 4 Tian Tan Xi Li, Beijing 100050, P. R. China

Tel: 86-13901158437; E-mail: lijun3021@aliyun.com

Table S1: Basic information of datasets from the Gene Expression Omnibus.

| Dataset ID | Platform | Organism | First author | Year | Region | Sample size (DM/N) | Type |
| --- | --- | --- | --- | --- | --- | --- | --- |
| GSE76364 | GPL19836 | RN | Takahara S | 2017 | Japan | 10/10 | mRNA |
| GSE76365 | GPL21257 | RN | Takahara S | 2020 | Japan | 10/10 | miRNA |

RN: Rattus norvegicus; DM: diabetes mellitus; N: normal.

Table S2: The primer sequences used

| Gene ID | Forward | Reverse |
| --- | --- | --- |
| rno-miR-6322 | CGCGTGTCTGTACCACATGG | AGTGCAGGGTCCGAGGTATT |
| rno-miR-674-3p | GCGCACAGCTCCCATCTCA | AGTGCAGGGTCCGAGGTATT |
| rno-miR-6324 | GCGTCAGTAGGCCAGACAGC | AGTGCAGGGTCCGAGGTATT |
| rno-miR-3557-3p | CGCGACACAGGACCTGGAGT | AGTGCAGGGTCCGAGGTATT |
| rno-miR-668 | TCACTCGGCTCGGCCC | AGTGCAGGGTCCGAGGTATT |
| rno-miR-3552 | CGAGGCTGCAGGCCCAC | AGTGCAGGGTCCGAGGTATT |
| rno-miR-207 | CGCTTCTCCTGGCTCTCCT | AGTGCAGGGTCCGAGGTATT |
| rno-miR-99a-3p | CGCGCAAGCTCGTTTCTATG | AGTGCAGGGTCCGAGGTATT |
| rno-miR-382-5p | GCGGAAGTTGTTCGTGGTG | AGTGCAGGGTCCGAGGTATT |
| rno-miR-185-3p | GCGCGTTTCCTCTGGTCC | AGTGCAGGGTCCGAGGTATT |
| rno-miR-212-5p | GCGACCTTGGCTCTAGACTGC | AGTGCAGGGTCCGAGGTATT |
| rno-miR-188-3p | GCGCTCCCACATGCAGG | AGTGCAGGGTCCGAGGTATT |
| rno-miR-219a-2-3p | GCGAGAATTGTGGCTGGAC | AGTGCAGGGTCCGAGGTATT |
| Smpd3 | CGTCGTCTGTGGAGATTTCA | GGTGAACAGGGAGTGTTGCT |
| Itga10 | TTCCCTCCATCCCTCACCTGTTC | GCATCTTCCACTCCCACCAGAAAC |
| Slc16a3 | ACCACTACTTCCAGTCGGCTACC | TCCCATCACTGTCCCGCAAGG |
| Xkr4 | CACACCTAGCACCGAACCACAC | CGGCGAGGGATGGGGAAGAG |
| Mx2 | TCCAATCCCACCACCTCCCAAG | GCCTGAGCATGTGAGTGTGAGTG |
| Gca | AATGCTGGCACAAGGAAGGAACC | CTGACCCTGGCACCATTGTATCTG |
| U6 | GCTTCGGCAGCACATATACTAAAAT | CGCTTCACGAATTTGCGTGTCAT |
| GAPDH | AGAAGGCTGGGGCTCATTG | AGGGGCCATCCACAGTCTTC |

Table S3: Significant differentially expressed miRNAs in type II diabetes mellitus

| miRNA | logFC | *P* value | Change |
| --- | --- | --- | --- |
| rno-miR-6322 | -1.64111 | 0.00385 | Down |
| rno-miR-674-3p | -1.26637 | 0.01118 | Down |
| rno-miR-668 | -1.09099 | 0.03718 | Down |
| rno-miR-3552 | -1.03907 | 0.04337 | Down |
| rno-miR-382-5p | -1.04002 | 0.04953 | Down |
| rno-miR-185-3p | -1.80529 | 0.01416 | Down |
| rno-miR-212-5p | -1.58212 | 0.01522 | Down |
| rno-miR-188-3p | -1.36589 | 0.0251 | Down |
| rno-miR-219a-2-3p | -1.3349 | 0.02713 | Down |
| rno-miR-6324 | 1.12791 | 0.01263 | Up |
| rno-miR-3557-3p | 1.13461 | 0.03057 | Up |
| rno-miR-207 | 1.05692 | 0.04606 | Up |
| rno-miR-99a-3p | 1.04558 | 0.04705 | Up |

Table S4: Key differentially expressed miRNAs and their target mRNAs in type II diabetes mellitus

| DEmRNA | logFC | predicted miRNA | *P* value | Change |
| --- | --- | --- | --- | --- |
| Smpd3 | -2.9576704 | rno-miR-207 | 0.01875144 | Down |
| Itga10 | -2.562503 | rno-miR-185-3p | 0.01254634 | Down |
| Slc16a3 | -2.0427927 | rno-miR-212-5p | 0.00494531 | Down |
| Xkr4 | -2.5792507 | rno-miR-6322 | 0.01072448 | Down |
| Mx2 | 3.0586312 | rno-miR-6322 | 0.02759349 | Up |
| Gca | 2.031708 | rno-miR-3557-3p | 0.00396622 | Up |

Figure S1. Blood glucose levels of rats at different timepoints after STZ injection.


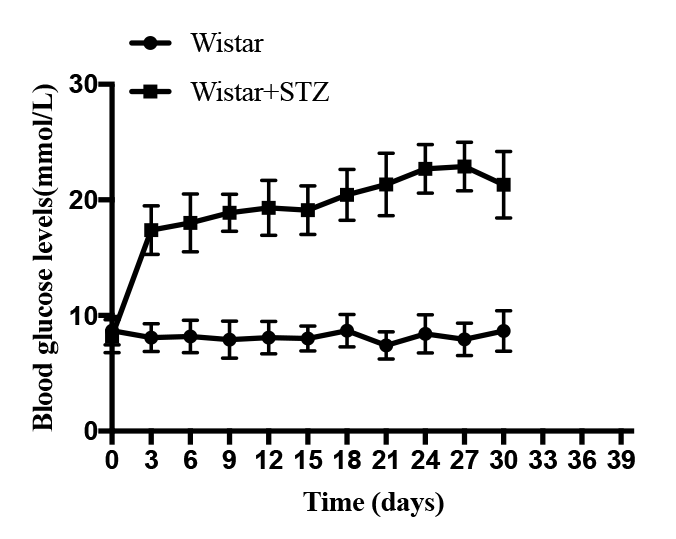


Table S1. Basic information of datasets from the Gene Expression Omnibus.

Table S2. The primer sequences used.

Table S3. Significant differentially expressed miRNAs in type II diabetes mellitus.

Table S4. Key differentially expressed miRNAs and their target mRNAs in type II diabetes mellitus.

Figure S1. Blood glucose levels of rats at different timepoints after STZ injection. After 3 days, the blood glucose levels can exceed 16.7 mmol/L. STZ, streptozotocin.
